# Effect of C-Terminal S-Palmitoylation on D2 Dopamine Receptor Trafficking and Stability

**DOI:** 10.1371/journal.pone.0140661

**Published:** 2015-11-04

**Authors:** Brittany Ebersole, Jessica Petko, Matthew Woll, Shoko Murakami, Kate Sokolina, Victoria Wong, Igor Stagljar, Bernhard Lüscher, Robert Levenson

**Affiliations:** 1 Department of Pharmacology, The Pennsylvania State College of Medicine, Hershey, Pennsylvania, United States of America; 2 Department of Biology, The Pennsylvania State University, University Park, Pennsylvania, United States of America; 3 Department of Biochemistry, University of Toronto, Toronto, Ontario, Canada; 4 Department of Molecular Genetics, University of Toronto, Toronto, Ontario, Canada; 5 Department of Biochemistry and Molecular Biology, The Pennsylvania State University, University Park, Pennsylvania, United States of America; 6 Center for Molecular Investigation of Neurological Disorders, The Pennsylvania State University, University Park, Pennsylvania, United States of America; Loyola University Chicago, Stritch School of Medicine, UNITED STATES

## Abstract

We have used bioorthogonal click chemistry (BCC), a sensitive non-isotopic labeling method, to analyze the palmitoylation status of the D2 dopamine receptor (D2R), a G protein-coupled receptor (GPCR) crucial for regulation of processes such as mood, reward, and motor control. By analyzing a series of D2R constructs containing mutations in cysteine residues, we found that palmitoylation of the D2R most likely occurs on the C-terminal cysteine residue (C443) of the polypeptide. D2Rs in which C443 was deleted showed significantly reduced palmitoylation levels, plasma membrane expression, and protein stability compared to wild-type D2Rs. Rather, the C443 deletion mutant appeared to accumulate in the Golgi, indicating that palmitoylation of the D2R is important for cell surface expression of the receptor. Using the full-length D2R as bait in a membrane yeast two-hybrid (MYTH) screen, we identified the palmitoyl acyltransferase (PAT) zDHHC4 as a D2R interacting protein. Co-immunoprecipitation analysis revealed that several other PATs, including zDHHC3 and zDHHC8, also interacted with the D2R and that each of the three PATs was capable of affecting the palmitoylation status of the D2R. Finally, biochemical analyses using D2R mutants and the palmitoylation blocker, 2-bromopalmitate indicate that palmitoylation of the receptor plays a role in stability of the D2R.

## Introduction

Palmitoylation is a posttranslational modification that can affect protein stability, membrane association, subcellular trafficking, desensitization, internalization, and signaling of a variety of proteins including G protein-coupled receptors (GPCRs; [1–7]). Palmitoylation typically involves covalent attachment of palmitate (saturated 16-carbon fatty acid) to proteins via an enzymatic reaction that is catalyzed, in humans, by a family of 23 known palmitoyl acyltransferases (PATs; [8]). PATs consist of four to six transmembrane domains and share a conserved aspartic acid-histidine-histidine-cysteine (DHHC) domain [9–14]. Palmitoylation enhances the hydrophobicity of proteins, thereby contributing to their membrane association, protein-protein interactions, and subcellular trafficking [15–17].

In the present study, we used bioorthogonal click chemistry (BCC), a sensitive non-isotopic labeling method [18, 19], to interrogate the palmitoylation status of the D2 dopamine receptor (D2R). D2Rs are GPCRs which, upon activation, couple to G_i_ proteins and lead to an inhibition of adenylate cyclase [18]. D2Rs are alternatively spliced and exist as two isoforms. The long isoform (D2L) contains a 29 amino acid insert in the third intracellular loop that is absent in the short isoform (D2S) [20–22]. This region is believed to be involved in specificity of G protein coupling. D2Rs are involved in a variety of cellular pathways, including those responsible for cognition, emotion, reward, and motor control [23, 24]. Dysfunction of D2R-mediated cellular signaling is associated with a number of human disorders including Parkinson’s disease, schizophrenia, and drug abuse [25]. The D2R is of particular pharmacological interest as it is the target for many antipsychotic drugs [26–28].

Previous studies, using baculovirus-infected insect Sf9 cells, have shown that the human D2Rs are palmitoylated [29, 30]. While no experimental information is currently available regarding the structural or functional significance of D2R palmitoylation in mammalian cells, a recent simulation study suggests that, like most Class A GPCRs, the D2R is palmitoylated on a cysteine residue at the terminus of helix 8 (Hx8). Hx8 is an amphipathic helical structure that connects the seventh transmembrane segment of many GPCRs to the remainder of the C-terminal cytoplasmic tail. For D2-like dopamine receptors (D2R, D3R, and D4R), the cysteine residue at the end of Hx8 is the terminal residue of the protein, and is known to constitute a PDZ binding site that is used for protein-protein interactions. Simulations demonstrated that palmitoylation of the terminal cysteine of D2R should increase membrane penetration of Hx8, rendering it less accessible to PDZ ligands such as GIPC [31].

To investigate the functional role of D2R palmitoylation, we have used BCC in conjunction with biochemical analysis of mutant D2Rs. Our results indicate that the D2R is palmitoylated in mammalian cells, and that the C-terminal cysteine residue, C443, is likely a major site of palmitoylation. We also identified several PATs (zDHHC3, zDHHC4, and zDHHC8) that interact with the D2R and are capable of affecting the palmitoylation state of the receptor. Biochemical analysis of D2R mutants revealed that palmitoylation of the receptor primarily affects its stability and trafficking to the plasma membrane.

## Materials and Methods

### Antibodies

Rabbit and mouse IgG were purchased from Santa Cruz Biotechnology, Inc. (Dallas, TX). Primary antibodies used in this study include rabbit and mouse anti-FLAG (Sigma-Aldrich Co., St. Louis, MO); mouse anti-myc, chicken anti-GAPDH, and rabbit anti-D2R (Millipore, Billerica, MA); rabbit anti-zDHHC8 (Santa Cruz Biotechnology); mouse anti-GM130 (BD Biosciences, San Jose, CA); mouse anti-GFP (Life Technologies, Carlsbad, CA), and rabbit anti-phospho-p44/42 MAPK (pERK) and rabbit anti-p44/42 MAPK (total ERK) (Cell Signaling, Beverly, MA). HRP-conjugated goat anti-mouse, goat anti-rabbit and donkey anti-chicken secondary antibodies were purchased from Jackson ImmunoResearch (West Grove, PA). Fluorescent secondary antibodies include Alexa Fluor 488-conjugated goat anti-rabbit, Alexa Fluor 555-conjugated goat anti-mouse, (Molecular Probes, Eugene, OR), and Cy3-conjugated donkey anti-mouse (Jackson ImmunoResearch).

### Yeast two-hybrid assays

To screen for novel D2R interacting proteins, we performed a modified split-ubiquitin membrane yeast two-hybrid (MYTH) screen as previously described [32–34]. Full-length human D2R cDNA in the bait vector pCCW-STE and a human fetal brain library in the prey vector pPR3-N (Dualsystems Biotech AG, Switzerland) were sequentially transformed into the *S*. *cerevisiae* reporter strain THY.AP4. Colonies that grew on quadruple drop out (SD) plates (-Trp/-Leu/-His/-Ade; Clontech Laboratories, Inc., Mountain View, CA) containing 3AT were picked and grown in liquid culture. cDNAs extracted from liquid cultures were sequenced and identities of the cDNAs determined by BLAST analysis.

To identify the sites of interaction between dopamine receptors and zDHHC proteins, cDNA sequences encoding the intracellular loops (IL) of the D2R (IL1, amino acids 61–70; IL2, amino acids 131–151; IL3, amino acids 214–373; IL3-NH_3_, amino acids 214–294; IL3-COOH, amino acids 295–372 and C-tail, amino acids 432–443), the D4R (IL2, amino acids 132–151), and the D5R (IL2, amino acids 137–158) were individually ligated into the pAS2-1 bait vector (Clontech). cDNAs encoding the zinc finger domains of zDHHC4 (amino acids 122–192) and zDHHC8 (amino acids 67–148) were cloned into the pACT2 prey vector. Bait and prey plasmids were co-transformed into the *S*. *cerevisiae* strain MaV103 and interactions were assayed for β-galactosidase activity via the nitrocellulose filter lift method [35].

### Cell culture, mutagenesis and immunofluorescence

Human embryonic kidney 293T (HEK-293T) cells were cultured in Dulbecco’s Modified Eagle Medium (DMEM) supplemented with 1% PenStrep and 10% fetal bovine serum. HEK-293 cells stably expressing FLAG-tagged D2L (HEK-D2L) were generously provided by Dr. Mark von Zastrow (University of California, San Francisco) [36]. Stable cell lines were maintained in DMEM supplemented with 10% fetal bovine serum and 400 μg/mL G418 (Invitrogen, Carlsbad, CA).

D2R mutants for palmitoylation analysis were generated by PCR mutagenesis, and the mutants and wild-type D2R constructs inserted into the pCMV-Tag 2 (FLAG) expression vector. GODZ (zDHHC3), zDHHC4, and zDHHC8 cDNAs were inserted into pEGFP (GFP for immunofluorescence) and pCMV-Tag 3 (myc for biochemical analysis) vectors. Transfections were performed using either CaPO_4_ [37] or Effectene transfection reagent (Qiagen, Valencia, CA).

For immunofluorescence, cells were grown on poly-lysine coated coverslips, washed with Dulbecco’s phosphate buffered saline (DPBS), fixed with 4% paraformaldehyde, permeabilized with 0.2% Triton X-100 in PBS containing 10% normal donkey serum (Jackson ImmunoResearch), and stained with the indicated antibodies. The coverslips were mounted onto glass slides using Prolong Gold Anti-Fade Mountant (Life Technologies). Fluorescent images were captured with a confocal laser-scanning microscope (Olympus FlowView FV300) or an inverted fluorescent microscope (Nikon Eclipse TE2000-S) and overlaid using Adobe Photoshop.

### Co-immunoprecipitation

HEK-D2L cells transiently expressing myc-tagged zDHHC4, GODZ, or zDHHC8 were lysed in sodium phosphate lysis buffer (100 mM sodium phosphate, pH 7.5, 150 mM NaCl, 1% Nonidet P-40) containing protease inhibitors (cOmplete MINI EDTA free, Roche). Immunoprecipitation was performed using Protein G Magnetic Sepharose beads (GE Healthcare, Little Chalfont, Buckinghamshire, UK) coated with 5 μL of indicated antibodies and 1 mg of crude cell lysates as described previously [19]. Eluted proteins were separated by SDS-PAGE, transferred to a PVDF membrane, and immunoblotted with indicated antibodies.

### 2-BP treatment and bioorthogonal click chemistry

Palmitoylation of cellular proteins was analyzed using bioorthogonal click chemistry (BCC). Labeling of cellular proteins with 15-hexadecynoic acid (15-HDYA) and click chemistry reactions were carried out as previously described [19]. Briefly, cells were incubated with 100 μM 15-HDYA for 24 hours, harvested, and lysed in a sodium phosphate lysis buffer containing protease inhibitors (cOmplete MINI EDTA free, Roche). In experiments where PAT activity was inhibited, cells were additionally treated with 100 μM 2-bromopalmitate (2-BP) for 24 hours before lysis. For palmitoylation assays in which quantitation was performed, lysate blots were first run to determine D2R expression levels for normalization. Equal amounts of D2Rs were then immunoprecipitated as described above. Magnetic beads were resuspended and treated with click chemistry reagents (TCEP, TBTA, TAMRA-azide) for 1 hour at room temperature. Samples were eluted with 4x loading dye (250 mM Tris-HCl pH 6.8, 40% glycerol, 8% SDS, 0.10% bromophenol blue, 20% β-mercaptoethanol) and incubated at room temperature for 15 minutes. Proteins were separated by SDS-PAGE and in-gel imaging performed using a Typhoon 9410 fluorescent imager (532/580 BP 30, GE Amersham). Imaged gels were rinsed with water, transferred to PVDF membranes, and immunoblotted with indicated antibodies. Blots were scanned using a back-lit scanner and quantified using ImageJ software. Palmitoylation levels were normalized to immunoprecipitated D2R and data subjected to a two-sided unpaired Student’s t-test. Gels assayed for the effects of hydroxylamine were first imaged as described above and then cut in half. One half was treated with water (control) and the other half was treated with 1M hydroxylamine (HAM) for 48 hours. After treatment the gel was reimaged on the fluorescent imager.

### Cell surface biotinylation assay

HEK-293T cells transfected with FLAG-tagged WT-D2R or ΔC443-D2R, or HEK-D2L cells treated with DMSO or 15-HDYA were washed with PBS and treated with 300 μg/mL EZ-Link Sulfo-NHS-SS-Biotin (Thermo Scientific, Rockford, IL) in PBS for 30 minutes at 4°C. Biotin solution was removed, cells were washed with 50 mM Tris, pH 7.4,150 mM NaCl and lysed in sodium phosphate lysis buffer as described above. Lysates were analyzed by Western blotting for D2R expression. Equal amounts of D2R (as determined by a preliminary total lysate blot) were incubated with 100 μL of NeutrAvidin Agarose Resin (Thermo Scientific) for 3 hours at room temperature with end-over-end mixing. Beads were washed 5x with PBS. Biotinylated proteins were eluted with 1x loading dye and analyzed via SDS-PAGE and Western blotting. Blots were scanned using a back-lit scanner. Individual bands were quantified using ImageJ software and pixel densities subjected to a two-sided unpaired Student’s t-test.

### RT-qPCR analysis of gene expression

HEK-293T cells transiently transfected with equal amounts (0.4 μg) of WT-D2R or ΔC443-D2R cDNAs or HEK-D2L cells transiently transfected with equal amounts (0.4 μg) of GODZ or GODZ (DHHS) cDNAs were used. Total RNA was isolated using TRIzol Reagent (Life Technologies). cDNAs were synthesized using the Transcriptor First Strand cDNA Synthesis kit (Roche Applied Science). RT-qPCR was performed on an Applied Biosystems by Life Technologies QuantStudio 12K Flex (Foster City, CA) system using primers for human D2DR (Hs00241436_m1, Life Technologies). Relative quantities of D2R expression were calculated using QuantStudio 12K Flex 1.2.2 software and the 2^-ΔΔC^_t_ analysis method using GAPDH (Hs99999905_m1, Life Technologies) as the endogenous control. These studies were conducted with the assistance of the Functional Genomics Core Facility of the PSU Division of Shared Research Resources.

## Results

### D2R is S-palmitoylated in mammalian cells

**Fig 1:**
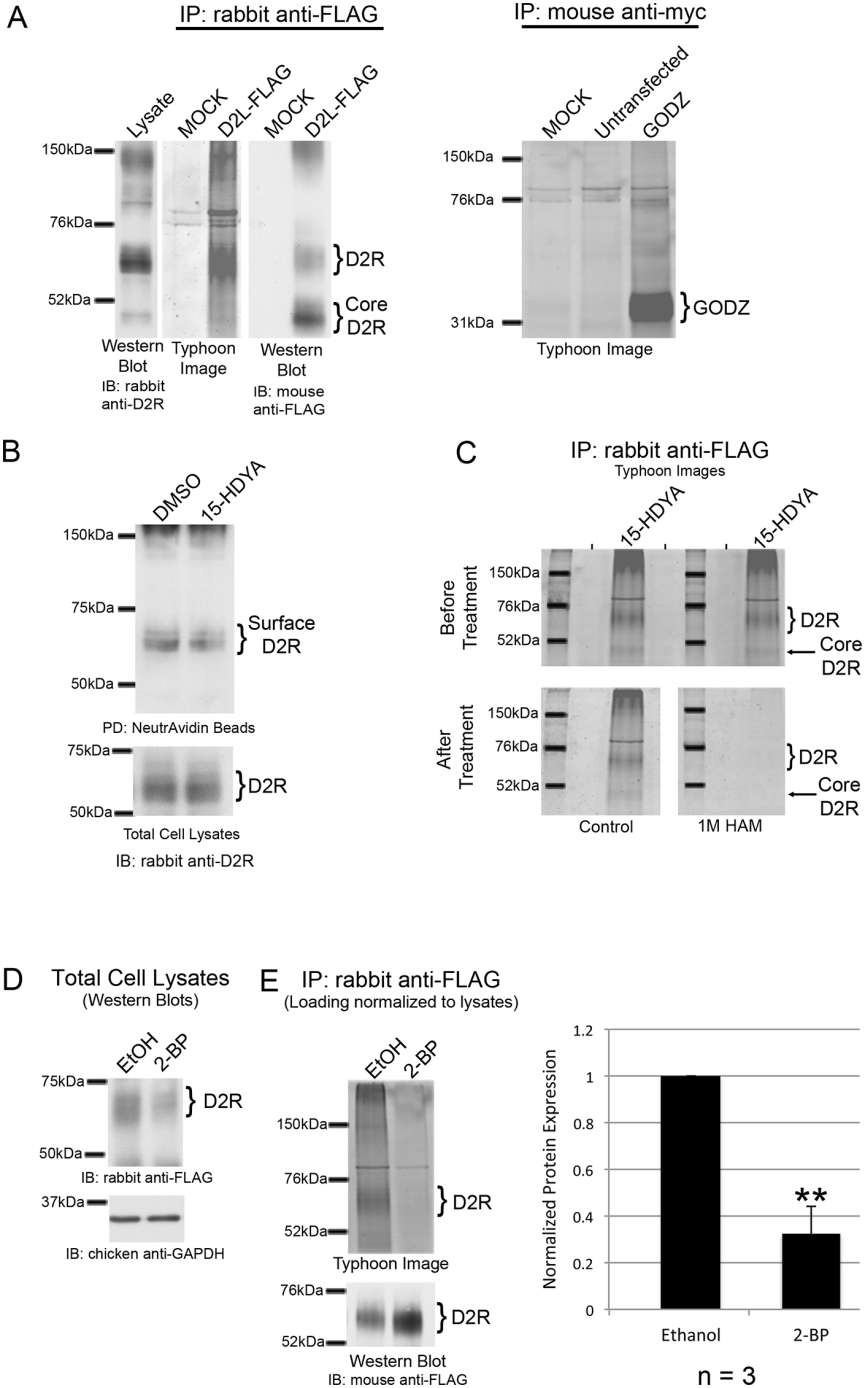
S-palmitoylation of D2L and D2S in mammalian cells. For panels A, C and E, D2R (from HEK-D2L cells) was labeled with 100 μM 15-HDYA followed by immunoprecipitation of either the D2R or GODZ, SDS-PAGE, and Typhoon imaging. For panels A and E, gels were further analyzed by Western blotting (IB: mouse anti-FLAG) for D2R. (A) Lysates from HEK-D2L cells were analyzed by SDS-PAGE and Western blotting for D2R (left, IB: rabbit anti-D2R). Palmitoylated D2L (middle, Typhoon Image) was compared to total receptor from IPs (right, IB: mouse anti-FLAG). A mock immunoprecipitation was performed with Protein G beads coated with non-specific rabbit IgG. Palmitoylation of GODZ was assayed and compared to a mock immunoprecipitation reaction and an immunoprecipitation of HEK-D2L cells that did not express myc-GODZ. (B) HEK-D2L cells treated with DMSO (0.2%) or 100 μM 15-HDYA were further treated with 300 μg/mL EZ-Link Sulfo-NHS-SS-Biotin. Biotinylated proteins were pulled down with NeutrAvidin Agarose Resin and analyzed via SDS-PAGE and Western blotting (top). Levels of biotinylated D2L were compared to the amount of receptor expressed in total cell lysates (bottom). (C) IPs of labeled D2L were run in duplicate on an SDS gel. The gel was scanned before (top) and after (bottom) treatment with either water (control) or 1 M hydroxylamine (HAM). (D) HEK-D2L cells were treated with 100 μM15-HDYA and either 100 μM 2-bromopalmitate (2-BP) or ethanol (0.2%) and receptor protein expression was analyzed by Western blotting (top). GAPDH was used as a loading control (bottom). (E) Normalized amounts of lysate from panel D were subjected to BCC. Brackets indicate bands corresponding to D2R that were used for quantitation. Levels of palmitoylated D2L (top) were normalized to the amount of immunoprecipitated receptor (bottom). The bar graph represents the average pixel density (as determined by ImageJ) from three separate experiments. Data was analyzed using a two-sided unpaired Student’s *t* test (expressed as ± SEM, *n* = 3).

Previous studies have shown that the D2 dopamine receptor (D2R) isoforms D2L and D2S are palmitoylated in baculovirus-infected insect Sf9 cells as measured by [3]H palmitate incorporation [29, 30]. Here we utilized BCC, a non-isotopic labeling method, to investigate palmitoylation of D2L in HEK-293 cells stably expressing the receptor (HEK-D2L). D2Rs were immunoprecipitated from HEK-D2L cells treated with 15-HDYA, a chemical probe that consists of palmitate with an omega terminal alkyne. The click reaction attaches a TAMRA-azide label to the probe, which can be imaged by in-gel fluorescence using a Typhoon scanner. Palmitoylated D2R was visualized as a broad band centered at ~70 kDa (Fig 1A, middle panel, Typhoon image) that corresponds to the glycosylated D2L species. Occasionally, a ~50 kDa protein corresponding to the D2L core protein was observed in palmitoylation assays (Fig 1C). It has been shown that the deglycosylated D1R, D2R, and D5R core proteins migrate with an apparent molecular weight of 44–46 kDa, whereas the glycosylated D2R species migrates between 65–70 kDa [38–40]. Both bands are detected via D2R antibody staining when the imaged gels are subjected to Western blotting (Fig 1A, right panel) as well as in total cell lysates (Fig 1A, left panel). Together, these results indicate that D2L can be palmitoylated in mammalian cells and that glycosylation of D2L may not be required for palmitoylation.

Aside from the expected banding pattern for D2L, D2R palmitoylation assays yield an additional band (occasionally a doublet) that migrates at ~76 kDa (Fig 1A, middle panel, Typhoon image). This band is also detected in BCC assays for other palmitoylated proteins including the mu opioid receptor and GODZ, a palmitoyl acyltransferase that is itself palmitoylated (Fig 1A, right panel, [19, 41, 42]). These results suggest that this ~76 kDa band represents a non-specific protein species.

In order to verify that 15-HDYA labeling did not interfere with receptor levels or proper plasma membrane expression, we performed a cell surface biotinylation experiment to quantitate the amount of D2L at the plasma membrane. Triplicate experiments revealed no change in total D2L levels in lysates or at the cell surface when comparing DMSO or 15-HDYA treatments (Fig 1B).

Of the three types of palmitoylation, S-, N- and O-, GPCR’s commonly contain S-linked palmitate attached to a cysteine residue via a thioester bond [7, 43, 44]. To test whether D2R palmitoylation is S-linked, we assessed the ability of hydroxylamine (HAM) to cleave the bond between D2L and the 15-HDYA label. HAM is a compound that specifically cleaves thioester bonds [14, 45, 46]. Two identical immunoprecipitation/BCC reactions were performed from a single dish of 15-HDYA-labeled HEK-D2L cells. As shown in Fig 1C (top panel), the two reactions contained equal amounts of palmitoylated D2L. Treatment with 1M HAM (pH 7.4) for 48 hours resulted in a drastic reduction in fluorescence intensity compared to soaking the gel in water (Fig 1C, bottom panel). This result indicates that the 15-HDYA probe is linked to D2L via a thioester bond.

As a secondary confirmation for S-linkage of the palmitate group on D2L, we examined the effect of 2-bromopalmitate (2-BP, a specific PAT inhibitor) on receptor palmitoylation in HEK-D2L cells. As mentioned earlier, PATs are the enzymes responsible for S-linked palmitoylation. Analysis of D2L protein expression in lysates of cells treated with 2-BP revealed decreased expression compared to control cells (Fig 1D). To account for this, the amount of cell lysate loaded into subsequent palmitoylation assays were adjusted to ensure equal D2R levels. 2-BP treatment significantly decreased the level of detectable palmitoylation of D2L compared to ethanol treated control cells (Fig 1E). This result supports the idea that D2Rs are S-palmitoylated and that PATs are responsible for promoting this protein modification.

### zDHHC4, GODZ, and zDHHC8 interact with D2R and increase levels of palmitoylated D2L

**Fig 2:**
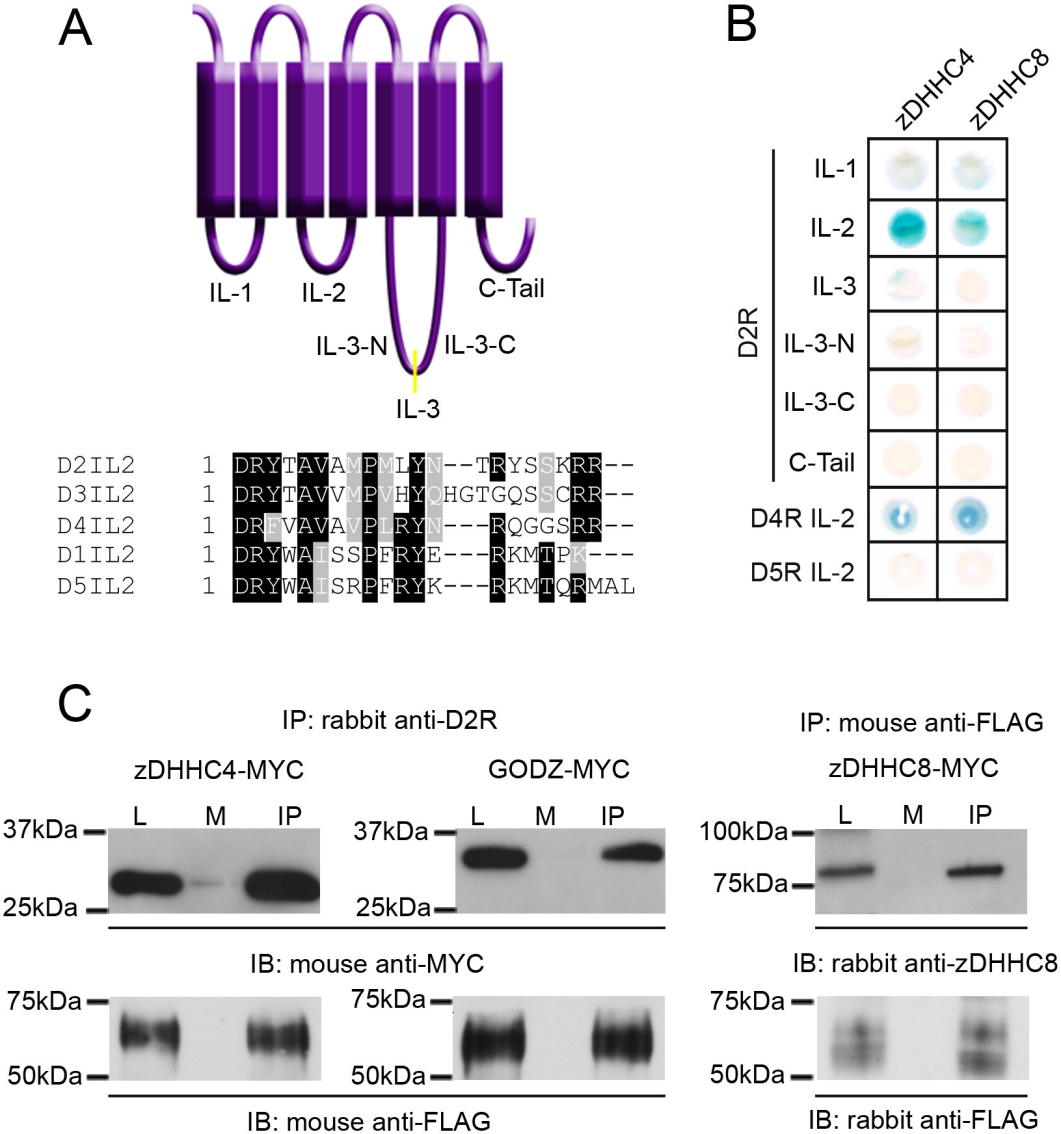
Interaction of D2L and D2S with candidate PATs in mammalian cells. (A) Schematic representation of D2L showing the intracellular portions of the receptor used as baits for directed yeast two-hybrid (Y2H) assay (top) and alignments of the second intracellular loops of all five dopamine receptors (bottom). (B) A Y2H assay was performed using intracellular portions of D2L, the second intracellular loop of D4R and the second intracellular loop of D5R (baits) with the zinc finger domains of zDHHC4 and zDHHC8 (preys). A positive interaction is indicated by the production of a blue yeast colony in the β-galactosidase assay. (C) FLAG-tagged D2L was immunoprecipitated from HEK-D2L cells with either rabbit anti-D2R or mouse anti-FLAG antibodies. Mock immunoprecipitations were performed with Protein G beads coated with non-specific rabbit or mouse IgG. Blots were probed with a mouse anti-myc or a rabbit anti-zDHHC8 antibody to test for co-immunoprecipitation (top) or mouse anti-FLAG or rabbit anti-FLAG to verify receptor immunoprecipitation (bottom). Lysate lanes (L) contain 5% of the total protein compared to the mock (M) and immunoprecipitation (IP) lanes.

We identified the PAT zDHHC4 as a potential D2L interactor in a MYTH screen in which the full length human D2R was used as bait to screen a human fetal brain cDNA library. To verify this interaction and to map the interaction site on D2L, we first performed a directed yeast two-hybrid assay in which the zinc finger domain of zDHHC4 was tested for interaction with each of the intracellular loops of D2L. We also tested the PAT zDHHC8 for interaction with D2L, because its gene maps to a schizophrenia susceptibility locus (22q11; [47, 48]). Additionally, a directed yeast two-hybrid assay was performed to test for interaction between the zinc finger domains of zDHHC4 and zDHHC8 and the second intracellular loop of the dopamine D4 (D4R) and dopamine D5 (D5R) receptors. The second intracellular loops of the dopamine D1 and D3 receptors were also assayed, however, due to their auto-activation of the GAL4 transcription factor they could not be analyzed using this method. Fig 2A shows a schematic representation of the intracellular portions of the D2L receptor as well as a sequence alignment of the second intracellular loop of all five dopamine receptors. As shown in Fig 2B, the DHHC-type zinc finger domains of zDHHC4 and zDHHC8 each interacted specifically with the second intracellular loop (IL-2) of D2R and D4R, but not D5R. These results suggest that these PATs may preferentially interact with the D2-like family of dopamine receptors.

We next utilized co-immunoprecipitation (co-IP) to analyze the interaction between full length PATs and the D2R within the context of mammalian cells. In addition to zDHHC4 and zDHHC8, we also examined the interaction between the D2R and zDHHC3 (GODZ), a PAT known for its role in palmitoylating other neuronal proteins [8, 47, 49]. As shown in Fig 2C, anti-D2R or anti-FLAG (IP lanes), but not IgG (Mock lane, M), were able to IP each of the PATs from HEK-D2L cells transiently transfected with any one of the myc-tagged PATs. Mouse anti-myc antibody detected bands migrating at the proper size in both lysate (L) and IP lanes for zDHHC4 and GODZ. The mouse anti-myc antibody was unable to detect expression of zDHHC8-myc. However, a rabbit antibody specific for zDHHC8 was able to detect a protein of the proper size in lysates and co-IPs from HEK-D2L cells transiently transfected with zDHHC8-myc but not in untransfected cell lysates (Fig 2C, top panels). Mouse or rabbit anti-FLAG antibody was used to verify receptor immunoprecipitation (Fig 2C, bottom panels). These results indicate that D2Rs can interact with zDHHC4, zDHHC8, and GODZ. Independent MYTH and co-IP experiments showed no interaction between D2R and zDHHC7 (SERZβ), suggesting that only certain PATs are capable of interacting with the D2R (S1 Fig).

**Fig 3:**
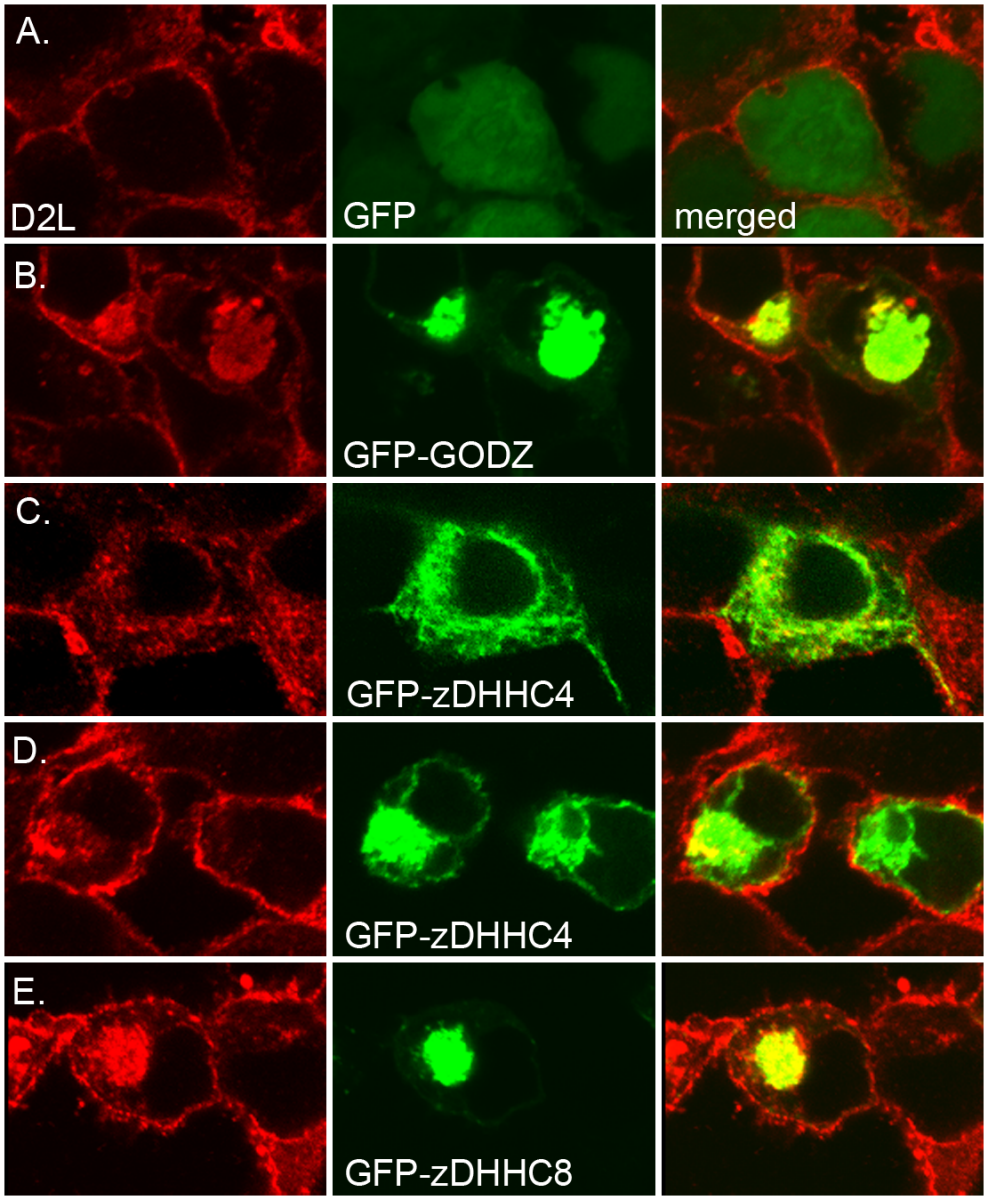
Co-localization of D2L and D2S with GFP-tagged PATs. D2 receptor constructs expressing D2L were co-expressed with GFP (A), GFP-GODZ (B), GFP-zDHHC4 (C, D), or GFP-zDHHC8 (E), followed by membrane permeabilization, fixation and immunofluorescent staining for D2L (red, left) and GFP (green, center) followed by confocal microscopy (right, co-localization in merged red and green channels evident in yellow). Two representative images are shown for low (C) and high (D) expression of GFP-zDHHC4.

To determine if the full-length D2R and each of the three PATs were located in similar subcellular compartments that would allow for D2R/PAT interaction, co-localization studies were performed. In these experiments, we used confocal microscopy to analyze the cellular localization of FLAG-tagged D2L receptors in conjunction with either GFP-tagged zDHHC4, GODZ, or zDHHC8 in HEK-D2L cells. As shown in Fig 3, all three PATs exhibited strong perinuclear expression, consistent with previous reports that these PATs accumulate in the Golgi [7, 8]. In addition to its presence in Golgi, zDHHC4 expression was also detected at the plasma membrane (Fig 3C). All three PATs showed significant overlap with D2L expression, indicating the potential for a direct interaction between these proteins in HEK-D2L cells. It is possible, however, that overexpression of D2L, in conjunction with overexpression of the PATs, may, in part, be responsible for the observed co-localization.

To determine whether zDHHC4, GODZ, and zDHHC8 affect D2R palmitoylation, we tested the effect of overexpressing each PAT on D2R palmitoylation levels. Because a majority of the 23 known PATs are expressed in HEK-293 cells and are known to compensate for one another, knockdown studies are not feasible [50].

**Fig 4:**
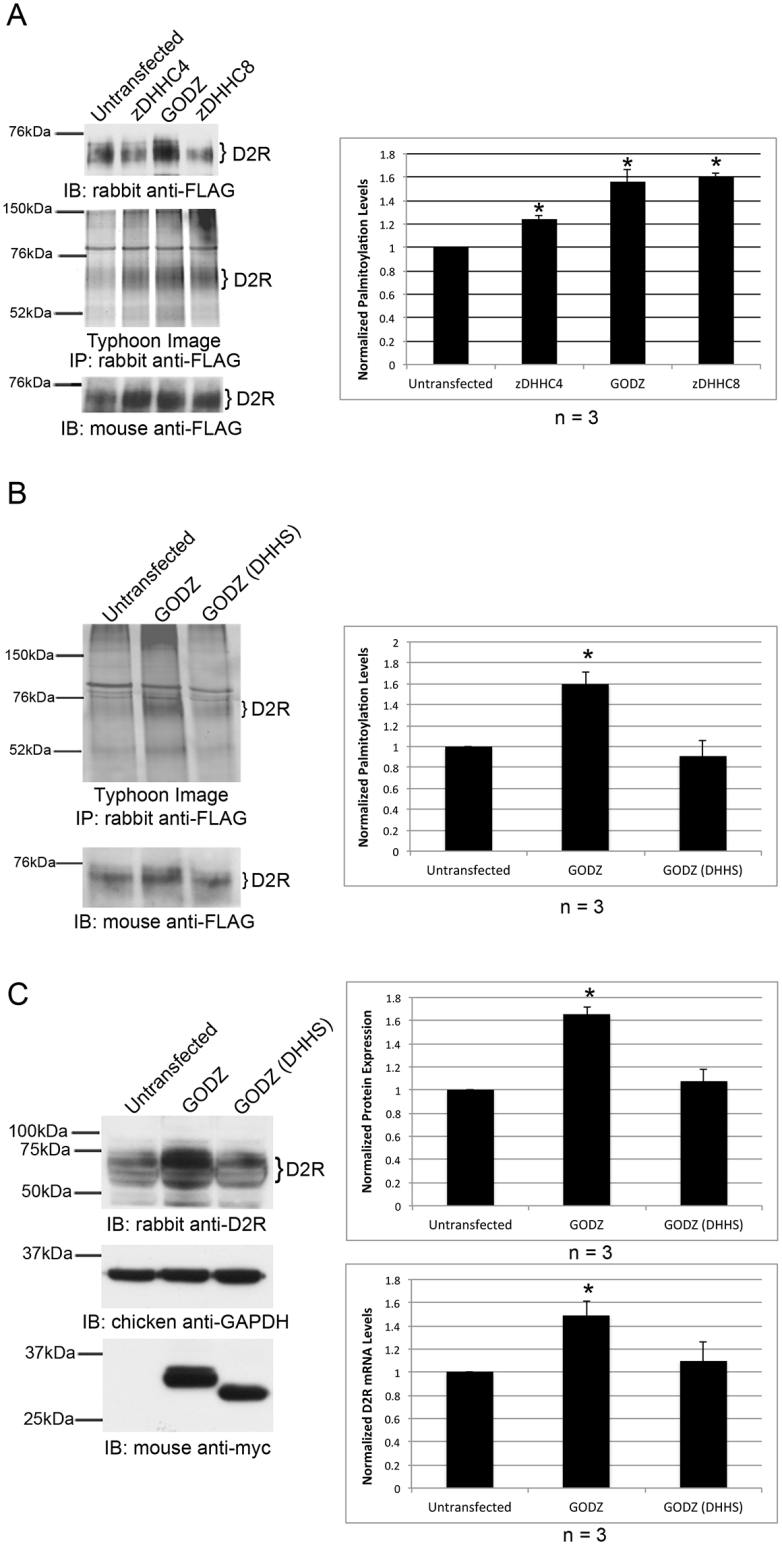
Overexpression of catalytically active PATs increase the amount of palmitoylated D2L. (A-C) myc-tagged PATs or a catalytically inactive variant of GODZ, GODZ (DHHS), were transiently expressed in HEK-D2L cells. Untransfected cells were used as a control. (A) Protein expression of D2R in lysates was analyzed via SDS-PAGE and Western blotting (top). Normalized amounts of 15-HDYA-labeled D2Ls were IP’d, and fluorescently imaged (middle). Total D2L in IPs was analyzed by Western blotting (bottom). (B) Normalized amounts of 15-HDYA-labeled D2Ls were IP’d, fluorescently imaged (top) and total D2L in IPs was analyzed by Western blotting (bottom). (C) Expression of D2R (top) and GODZ or GODZ (DHHS) (bottom) in lysates was analyzed via SDS-PAGE and Western blotting. Quantitation of receptor expression was normalized to GAPDH expression for protein (top graph) and mRNA (bottom graph). D2R mRNA expression levels in cells overexpressing GODZ or GODZ (DHHS) were determined by RT-qPCR and normalized to untransfected levels. (A-C) Brackets indicate bands corresponding to D2R that were used for protein quantitation. Levels of palmitoylated D2L were normalized to the amount of IP’d receptor (A & B). Bar graphs represent the average pixel density (as determined by ImageJ) from three experiments. Data was analyzed using a two-sided unpaired Student’s *t* test (expressed as ± SEM, *n* = 3, *P < 0.05).

For this reason, overexpression of PATs in cultured cells has commonly been used to assess the substrate specificity of a variety of different PATs [49, 51–53]. For this experiment, myc-tagged zDHHC4, GODZ, and zDHHC8 were separately transfected into HEK-D2L cells and expression of D2Rs in cell lysates was analyzed by Western blotting (Fig 4A, top). Normalized amounts of D2Rs were subjected to BCC palmitoylation assays. Palmitoylated D2R levels (Fig 4A, middle) were quantitated and normalized to the amount of immunoprecipitated D2R (Fig 4A, bottom). Overexpression of zDHHC4, GODZ, and zDHHC8 produced an increase in D2R palmitoylation of 24%, 56%, and 60%, respectively, compared with untransfected cells. To demonstrate that this effect required PAT activity, we compared the levels of palmitoylated D2R in cells overexpressing wild-type GODZ or a catalytically inactive GODZ in which the cysteine in the DHHC domain was mutated to serine (GODZ (DHHS)). This catalytically inactive mutant is incapable of palmitoylating the known GODZ target NR2A [6]. As shown in Fig 4B, overexpression of GODZ produced a 60% increase in the amount of palmitoylated D2R, whereas the catalytically inactive GODZ (DHHS) mutant had no effect. Taken together, these results suggest that zDHHC4, GODZ, and zDHHC8 all promote palmitoylation of D2R in transfected HEK-D2L cells.

In performing these experiments, we noticed that overexpression of GODZ resulted in a general increase in D2R protein expression, while overexpression of GODZ (DHHS) had no effect (Fig 4C, top panel & graph). RT-qPCR analysis revealed a statistically significant increase in mRNA levels of D2R upon overexpression of GODZ but not GODZ (DHHS) suggesting that this change is, at least in part, due to changes in transcript levels (Fig 4C, bottom graph). However, this does not rule out the possibility that GODZ overexpression may result in altered D2R protein stability.

### Cysteine residue C443 is a site of D2L palmitoylation

**Fig 5:**
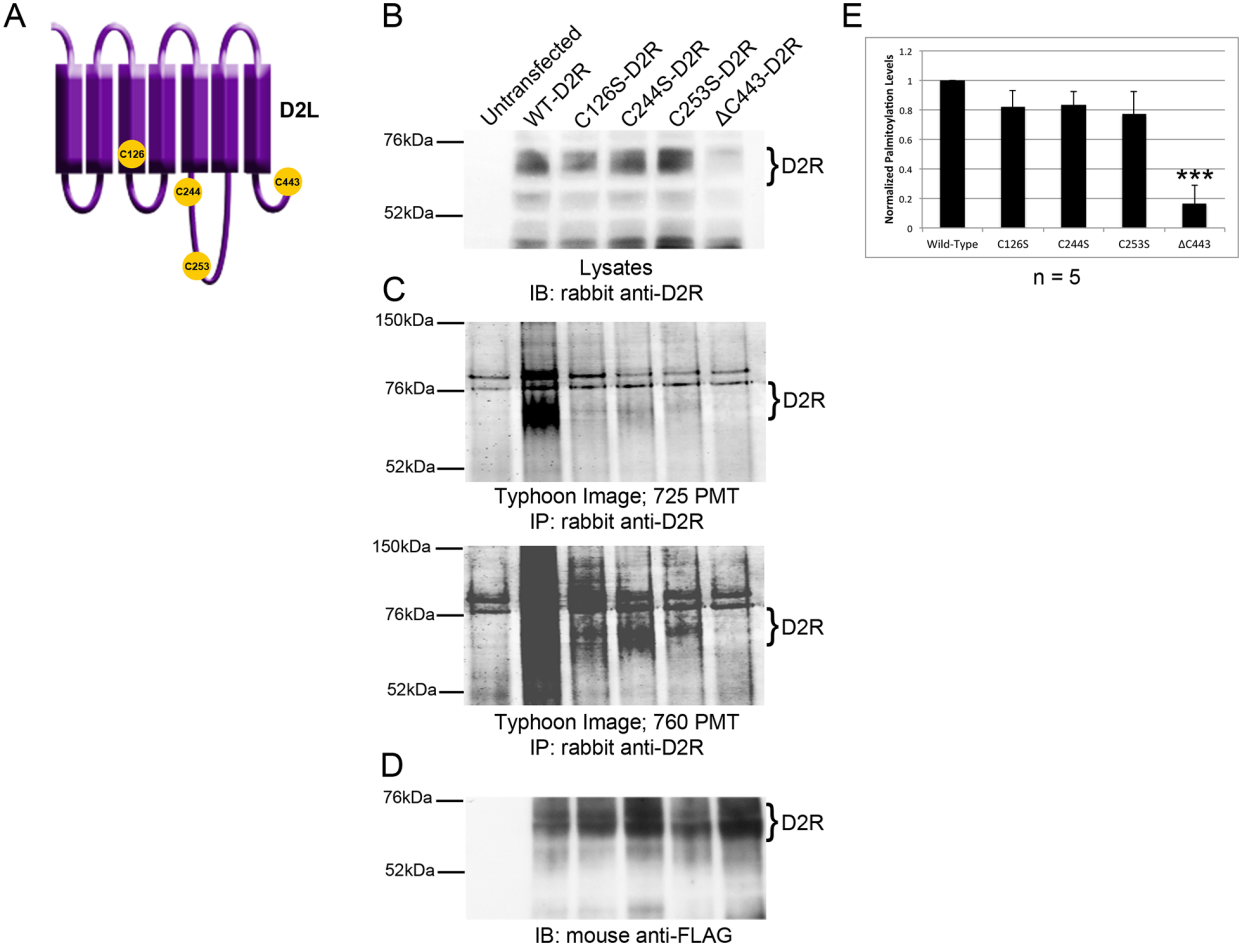
C443 is the major site of palmitoylation of the D2R. (A) Schematic representation of D2L showing the location of cysteine residue mutations. (B) FLAG-tagged WT-D2R or D2R mutants were transiently expressed in HEK-293T cells and expression in cell lysates was analyzed by Western blotting. Untransfected cells served as controls. (C) Normalized amounts of 15-HDYA-labeled D2Rs were IP’d and fluorescently imaged at 725 PMT (top) and 760 PMT (bottom). (D) Total D2R from IPs was analyzed by Western blotting. (E) Levels of palmitoylated D2Rs were normalized to the amount of IP’d receptor. Brackets indicate bands corresponding to D2R that were used for quantitation. The bar graph represents the average pixel density (as determined by ImageJ) from five separate experiments. Data were analyzed using a two-sided unpaired Student’s *t* test (expressed as ± SEM, *n* = 5, ***P < 0.001).

To determine the site of D2L palmitoylation, three cysteine to serine mutations (C126S, C244S, C253S) and a deletion mutation removing the C-terminal cysteine residue (ΔC443) were separately introduced into the D2R (Fig 5A). C126 was chosen for mutational analysis, because it is located just within the third transmembrane domain of the D2R, a known site for the palmitoylation of other proteins [54]. Additionally, we tested C244 and C253 as potential palmitoylation sites because they are cysteine residues present in D2L but absent in D2S. Finally, C443 was chosen because it is the only cysteine residue within the C-terminal tail of D2L. This site is of interest, since previous studies have shown that many GPCRs are palmitoylated in their C-terminal tails at the end of helix 8 [7, 31, 43, 44]. Using the online palmitoylation predictor program, PalmPred, C433 was the only cysteine predicted to be palmitoylated in the D2R polypeptide [55].

Each cysteine mutant construct, as well as a WT-D2R cDNA, was transiently transfected into HEK-293T cells and the expressed D2Rs analyzed for palmitoylation using 15-HDYA and BCC. The amount of lysate loaded into each BCC reaction was normalized to ΔC443-D2R expression, as this construct was expressed at lower levels than WT-D2R and the other three mutant constructs (Fig 5B). Palmitoylation of the WT-D2R and mutant D2Rs are shown at two different photomultiplier tube (PMT) values in Fig 5C. Palmitoylation levels were normalized to the amount of immunoprecipitated receptor (Fig 5D). Statistical analysis of five separate experiments showed that none of the cysteine to serine substitutions significantly reduced palmitoylation of the D2R, whereas the ΔC443 mutant showed an 83% decrease in the amount of palmitoylated receptor compared to WT-D2R (Fig 5E). Even at a high PMT (760), an exposure at which WT-D2R palmitoylation is highly saturated, there is little detectable palmitoylated ΔC443-D2R. These results indicate that C443 is the predominant site of D2R palmitoylation.

### Palmitoylation of cysteine residue C443 is required for D2R trafficking to the cell surface and protein stability

**Fig 6:**
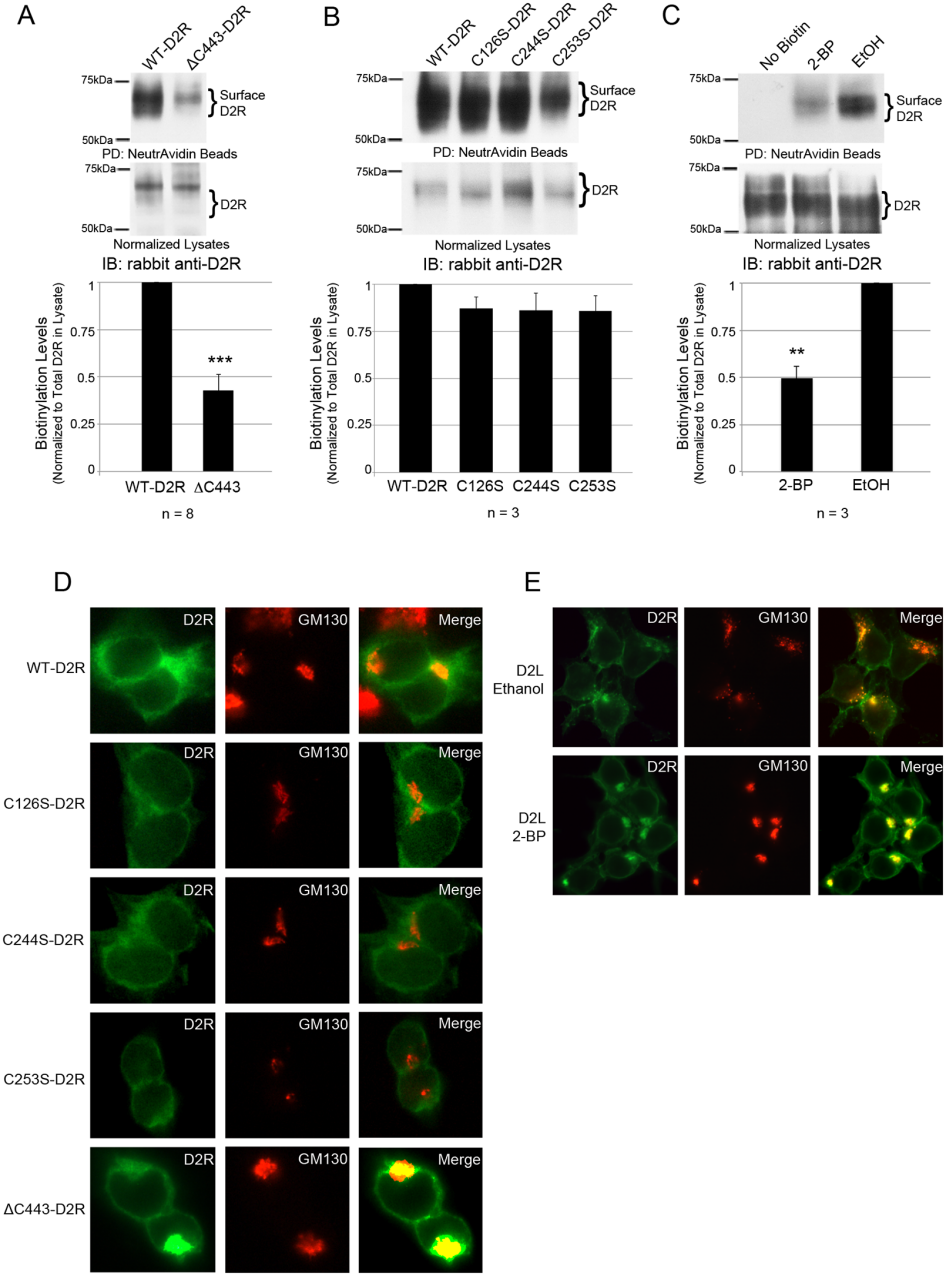
Deletion of C443 results in decreased cell surface expression of the D2R. Transient expression of (A) FLAG-tagged WT-D2R or ΔC443-D2R in HEK-293T cells, or (B, D) FLAG-tagged WT-D2R, C126S-D2R, C244S-D2R or C253S-D2R. (C, E) HEK-D2L cells treated with 2-BP or ethanol. (A-C) Cells were treated with 300 μg/mL EZ-Link Sulfo-NHS-SS-Biotin. Biotinylated proteins were pulled down with NeutrAvidin Agarose Resin and analyzed via SDS-PAGE and Western blotting (top). Levels of biotinylated D2L were normalized to the amount of receptor expressed in the lysate (middle). The bar graph represents the average pixel density (as determined by ImageJ) from either eight (A) or three (B, C) separate experiments. Data were analyzed using a two-sided unpaired Student’s *t* test (expressed as ± SEM, *n* = 8 (A), *n* = 3 (B, C), **P<0.01, ***P < 0.001). (D, E) Cell membranes were first permeabilized, then cells were fixed and stained for D2R (green, left) and the Golgi marker GM130 (red, center) and analyzed by fluorescence microscopy (right, Merged image). Images were overlaid using Adobe Photoshop.

It has been shown for several GPCRs, including the D1 dopamine receptor, vasopressin V2 receptor, TSH receptor, histamine H2 receptor and δ-opioid receptor, that palmitoylation is required for proper receptor cell surface expression [43]. To test whether palmitoylation of cysteine residue C443 plays a role in D2R plasma membrane expression, we employed a cell surface biotinylation assay to quantitate the expression of WT-D2R and ΔC443-D2R at the cell surface of transfected HEK-293T cells. Due to the varying expression levels of the D2R constructs, and the effect of 2-BP treatment on D2R protein expression, the amount of lysate added to pulldown reactions was normalized to a lysate blot (not shown). This was done to ensure equal receptor loading. As shown in Fig 6A, the cell surface expression of ΔC443-D2R was reduced by 57% compared to WT-D2R. In contrast to ΔC443-D2R, the C126S, C244S, and C253S mutations had no significant effect on cell surface expression when compared to receptor expression in normalized lysates (Fig 6B). To confirm that the reduced plasma membrane accumulation of the ΔC443-D2R mutant was due to the lack of palmitoylation and not the deletion itself, we also analyzed cell surface expression of the wild-type D2L in HEK-D2L cells treated with 2-BP. As shown in Fig 6C, cells treated with 2-BP showed a 51% reduction in plasma membrane localization of the D2R compared to control cells. Taken together, the results from these experiments are consistent with the idea that palmitoylation of the D2R C-terminal cysteine plays an important role in regulating cell surface expression of the D2R.

We next wished to determine whether decreased cell surface expression of the ΔC443-D2R mutant was a result of impaired trafficking to the plasma membrane. We used immunofluorescence to analyze the cellular localization of the ΔC443-D2R mutant. As shown in Fig 6D, WT-D2R and the C126S, C244S and C253S mutant D2Rs were localized predominantly at the plasma membrane. In contrast, the ΔC443-D2R mutant showed increased perinuclear staining that significantly overlapped with that of the cis-Golgi-specific marker GM130 (Fig 6D, bottom panel). To determine whether the cytosolic accumulation of ΔC443-D2R (in what appears to be predominantly Golgi) was due to its reduced palmitoylation, we analyzed the cellular localization of the D2R in HEK-D2L cells treated with the palmitoylation inhibitor 2-BP. In control cells, D2R expression was localized primarily to the plasma membrane. In contrast, in cells treated with 2-BP, the D2R showed a marked increase in expression that also overlapped with the GM130 cis-Golgi marker when palmitoylation was inhibited (Fig 6E). These results suggest that palmitoylation plays a key role in the trafficking of the D2R from the Golgi to the plasma membrane.

**Fig 7:**
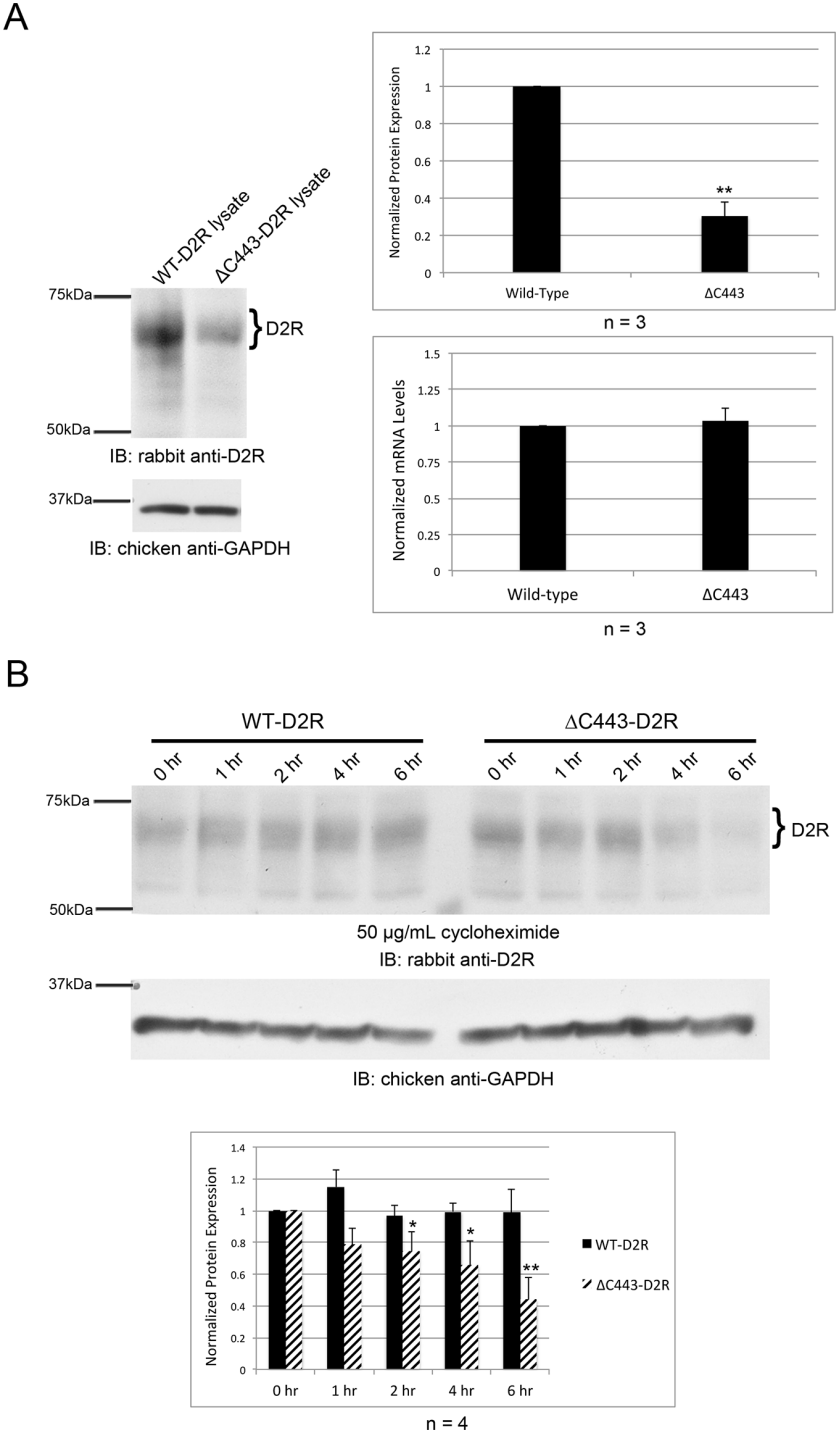
Deletion of C443 affects stability of the D2R. (A, B) FLAG-tagged WT-D2R or ΔC443-D2R cDNAs were transiently expressed in HEK-293T cells. Quantitation of receptor expression was normalized to GAPDH expression for protein and mRNA. (A) Proteins were separated by SDS-PAGE and analyzed by Western blotting (left). WT-D2R and ΔC443-D2R mRNA expression levels were determined by RT-qPCR and normalized to wild-type levels (bottom graph). The top bar graph represents the average pixel density (as determined by ImageJ) from three separate experiments. All data were analyzed using a two-sided unpaired Student’s *t* test (expressed as ± SEM, *n* = 3, **P<0.01). (B) Cells were treated with 50 μg/mL cycloheximide for the indicated times. Proteins were separated by SDS-PAGE and analyzed by Western blotting (top). The bar graph represents the average pixel density (as determined by ImageJ) from four separate experiments. Data were analyzed using a two-sided unpaired Student’s *t* test (expressed as ± SEM, *n* = 4, *P < 0.05, **P<0.01).

Palmitoylation of a variety of substrates has previously been shown to affect protein stability. As mentioned in the previous section, ΔC443-D2R protein expression was reduced in comparison to WT-D2R, even though equal amounts of DNA were transfected. To determine whether palmitoylation has an effect on the stability of the D2R polypeptide, HEK-293T cells were transiently transfected with either WT-D2R or ΔC443-D2R, and the expression levels of wild-type and mutant D2Rs analyzed by Western blotting and RT-qPCR. As shown in Fig 7A, the level of protein expression of the ΔC443-D2R mutant was reduced by 70% compared to the WT-D2R, with no significant difference in mRNA levels for either transcript. To determine whether the reduction in ΔC443-D2R expression levels was the result of reduced stability or synthesis, cells were treated with 50 μg/mL cycloheximide for various times to block protein synthesis, and D2R expression levels determined by Western blotting. By 2 hours of cycloheximide treatment, we observed a significant decrease in ΔC443-D2R protein expression, whereas WT-D2R expression remained unchanged for up to 6 hours of cycloheximide treatment (Fig 7B). Together, our experimental data support the notion that palmitoylation is required for proper trafficking and stability of the D2R.

## Discussion

We have used BCC, a non-isotopic labeling method utilizing 15-HDYA as a lipid probe, to interrogate the palmitoylation status of the D2 dopamine receptor. Our results are consistent with the following conclusions. First, our data indicate that the D2L is S-palmitoylated. Second, using a mutational approach, we identified C443, the terminal amino acid of the D2R polypeptide, as a major site of palmitoylation. Third, we identified three palmitoyl acyltransferases (PATs), zDHHC3 (GODZ), zDHHC4, and zDHHC8, each of which interacts with the D2R polypeptide and affects palmitoylation of the receptor. Fourth, we demonstrate that palmitoylation of the D2R on C443 is required for proper trafficking of the receptor to the plasma membrane and contributes to the stability of the receptor either in transit or once it reaches the cell surface.

These interpretations are based on the following observations. The data presented in Fig 1 indicates that BCC, in combination with immunoprecipitation, is an effective method for detecting D2R palmitoylation. Palmitoylation of the D2R appears to be primarily S-linked, since hydroxylamine, which specifically cleaves thioester bonds at S-linked sites [14, 45, 56, 57], effectively removed palmitate moieties from the D2R. Further, palmitoylation of the D2R was blocked by 2-BP, a specific inhibitor of the PATs responsible for S-linked palmitoylation [7, 43, 58–60]. Although earlier studies [29, 30] indicated that the D2R was a palmitoylated protein, these studies relied on D2R overexpression in baculovirus-infected insect Sf9 cells. Here, we show for the first time that the D2R is palmitoylated in a mammalian cell system, providing support for the idea that palmitoylation of the D2R has physiological relevance in mammalian species.

Amongst the D2-like dopamine receptors, there is a high amount of sequence homology within the second intracellular loop. Because this is the site of PAT interaction with D2R, we hypothesized that PATs would similarly bind to the second intracellular loop of D3R and D4R. We did, in fact, show through a yeast two-hybrid assay that D4R was able to associate with zDHHC4 and zDHHC8 through its second intracellular loop. We were unable to use this approach to test interaction with the D3R, as its second intracellular loop auto-activated this system. In contrast, the second intracellular loop of the D5R did not appear to associate with any of these PATs. There are several amino acids that are not conserved between the D1-like and D2-like families of dopamine receptors. It would be interesting to sequentially mutate these amino acids to determine whether they are essential for PAT binding.

Many GPCRs, including rhodopsin and the β_1_-adrenergic receptor, are known to be palmitoylated on cysteine residues located at the end of helix 8 within the C-terminal domains of the respective polypeptides [61, 62]. In the current study, we demonstrate that deletion of the C-terminal cysteine residue virtually eliminates detectable palmitoylation of D2R while three other cysteine to serine mutations, within the D2R, had much less of an effect on the palmitoylation state of D2R. This is in agreement with a recent simulation study that predicted palmitoylation of the D2R occurs on the C-terminal cysteine based on its proximity to helix 8. This study concluded that palmitoylation of this residue would result in insertion of the helix 8 backbone into the plasma membrane [31]. Sequence analysis indicates that there are 8 additional cysteine residues in the D2R that could represent potential sites for palmitoylation. However, none of these cysteine residues are located within any of the known consensus regions for protein palmitoylation. Our results are therefore most consistent with the idea that C443 represents the primary site of S-palmitoylation in the D2R.

All D2-like dopamine receptors (D2R, D3R, D4R) contain a C-terminal cysteine which is part of a type III PDZ-binding motif. This suggests the possibility that the site and function of palmitoylation in these receptors are conserved. Interestingly, the simulation study discussed above suggests that palmitoylation of the D2R on C443 would affect binding of PDZ-domain containing proteins, such as GIPC. GIPC has been shown to interact with the C-terminal cysteine of D2R and D3R (but not D4R) and prevent their lysosomal degradation. It is therefore possible that the results obtained with the C443 mutant could be due to disrupted interaction with GIPC. However, 2-BP treatment should not disrupt the GIPC binding site and provided similar results to C443 mutant studies. It will be of interest, then, to determine whether palmitoylation plays a role in regulation of GIPC binding.

**Fig 8:**
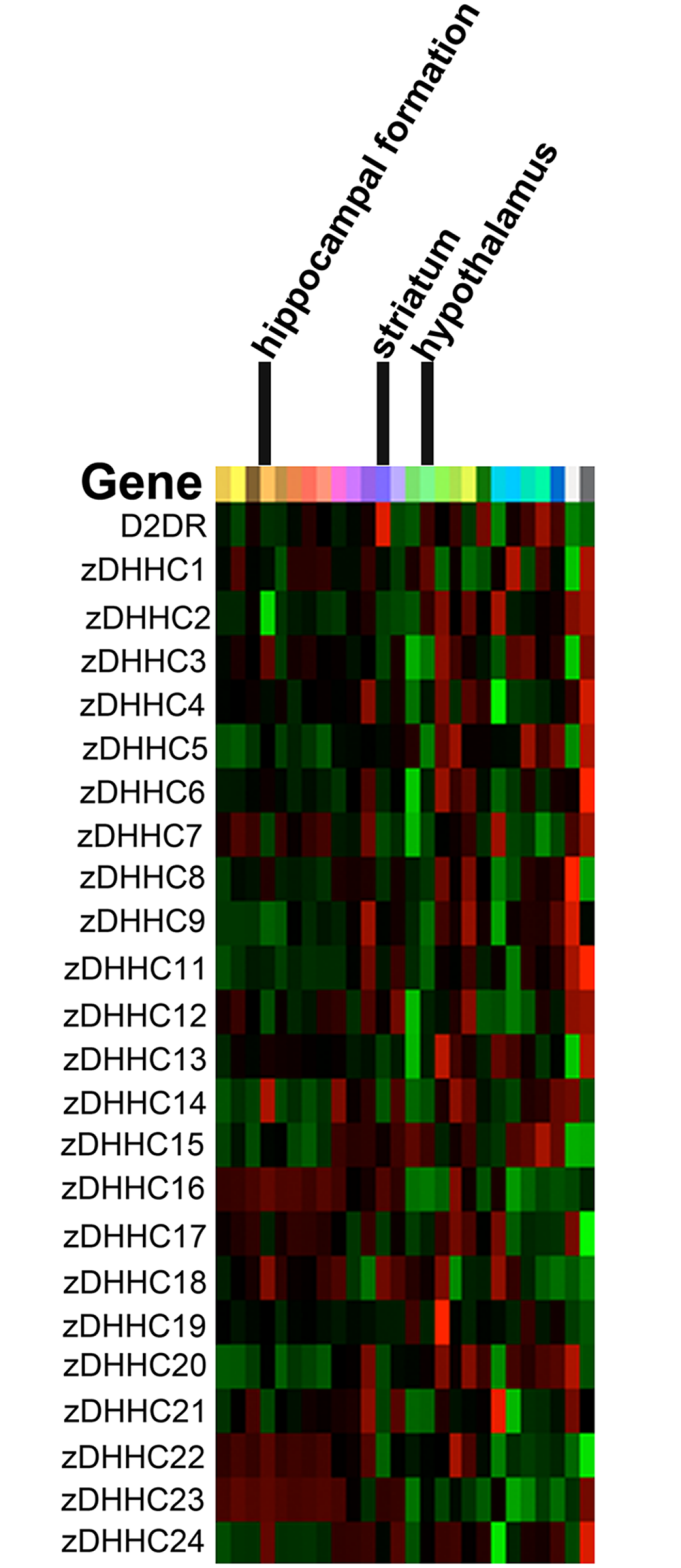
Expression profiles of 23 PATs in human brain. Heat map showing gene expression of D2R and the 23 PATs in the human brain. The heat map shows expression of these genes in one individual. 2014 Allen Institute for Brain Science. Allen Human Brain Atlas [Internet]. Available from: http://human.brain-map.org/.

Using a combination of yeast two-hybrid screening, co-immunoprecipitation, and co-localization studies, we identified zDHHC3 (GODZ), zDHHC4, and zDHHC8 as PATs that interact with the D2R. Overexpression of PATs in cultured cells has been used previously to assess PAT enzymatic activity [49, 51–53]. Overexpression of zDHHC4, zDHHC8, and GODZ all led to a statistically significant increase in the amount of palmitoylated D2R, whereas a catalytically inactive form of GODZ did not. This suggests that each of the three PATs we tested are capable of palmitoylating the D2R, at least in the context of transfected mammalian cells. However, the potential interaction of these (and other) PATs with the D2R *in vivo* is not known. The specificity of the interactions will depend in large part on whether the D2R and a particular PAT are co-expressed within the same cell type or cellular compartment within the nervous system. A study performed to assess intracellular localization and tissue-specific distribution of human PATs revealed that while some PATs have highly ubiquitous expression patterns, others show a tissue-specific expression pattern [63]. Additionally, within the brain, different PATs are localized within different brain regions. The expression pattern of PAT mRNAs throughout the human brain is shown in Fig 8 (reproduced with permission from the Allen Human Brain Atlas). The 23 known human PATs exhibit vastly different expression patterns throughout the brain, lending support to the idea that the specificity of PAT interactions with the D2R most likely results from differential PAT expression in different brain regions.

A comparison of the data in Figs 5 and 6 indicates that the primary function of D2R palmitoylation is to regulate proper trafficking of the receptor to the plasma membrane, and contribute to the stability of the receptor either in transit or after it reaches the cell surface. Deletion of C443, or inhibition of palmitoylation, resulted in a significant decrease in D2R expression at the cell surface, and a concomitant increase in receptor levels in what appears to be the Golgi. The view that palmitoylation may serve an important role in D2R trafficking is consistent with the data reported in several other systems [43]. We did attempt to determine whether this effect on D2R surface expression translated to a change in D2R signaling upon drug stimulation. However, signaling through the receptor, either via the MAPK or adenylyl cyclase pathways, appeared unaffected by blocking D2R palmitoylation (S2 Fig). This is somewhat surprising in light of the decreased cell surface expression exhibited by non-palmitoylated D2R. However, the amount of D2R that is able to reach the plasma membrane in this overexpression system may be enough to saturate the signaling response through pERK and cAMP, thus obscuring the effect of palmitoylation on signaling through the D2R.

Deletion of C443 also resulted in decreased levels of D2R protein expression compared to WT-D2R. ΔC443-D2R and wild-type receptor exhibited no detectable differences in mRNA expression, whereas the mutant protein showed an increased rate of degradation compared to WT-D2R. This suggests that palmitoylation also contributes to the overall stability of the D2R polypeptide within the cell.

The fact that zDHHC8 interacts with and can contribute to palmitoylation of the D2R bears further consideration. zDHHC8 in humans is encoded by the *ZDHHC8* gene located on chromosome 22q11, a locus associated with susceptibility to schizophrenia [64, 65]. Microdeletions of the 22q11.2 locus give rise to DiGeorge syndrome. Children with this microdeletion exhibit a wide spectrum of cognitive defects [66–68], and ~30% of them develop schizophrenia or schizoaffective disorder in adolescence or early adulthood [69, 70]. Previous studies have shown that zDHHC8-knockout mice exhibit deficits in several endophenotypes associated with schizophrenia including prepulse inhibition, a decrease in exploratory activity in a new environment, and a decreased sensitivity to the locomotor stimulatory effects of the psychomimetic drug dizocilpine (MK801). Additionally, a mouse model carrying a chromosomal deficiency syntenic to the 22q11.2 microdeletion experienced decreased dendritic spine and glutamatergic synapse density in addition to diminished dendritic growth of hippocampal neurons. Reintroduction of enzymatically active zDHHC8 protein prevented these endophenotypes [71]. These altered behaviors revealed a connection between impaired palmitate modification of neuronal proteins and the psychiatric phenotypes associated with microdeletions of chromosome 22q11. It is therefore of considerable interest that our data indicates that the D2R, a principal target of the antipsychotic drugs used to treat schizophrenia, may represent a substrate for palmitoylation by zDHHC8. Previous studies have shown that several synaptic proteins are substrates of zDHHC8 [8], which supports the idea that palmitoylation via this PAT could be important for regulating neuronal function. Based on our data, it is tempting to speculate that the cognitive and emotional deficits associated with the 22q11 deletion may result, at least in part, from failure to properly palmitoylate the D2R in individuals carrying this chromosomal abnormality. Further experiments should be performed to evaluate this possibility, such as analyzing levels of palmitoylated D2R in zDHHC8 knockout mice or mouse models of other D2R-related diseases.

## Supporting Information

S1 Fig D2R interacts with zDHHC4 but not zDHHC7 (SERZβ). (A) MYTH assay in which yeast cells expressing human D2L were transformed with the following prey constructs: Fur4-NubG (negative control), Fur4-NubI (positive control), NubG-zDHHC4, and NubG-SERZβ. A positive interaction between D2L and NubG-zDHHC4 was detected, however, no interaction was observed with D2L and NubG-SERZβ, indicating that zDHHC4 is a D2L-specific interacting protein (blue yeast colonies). (B) HA-tagged D2L was immunoprecipitated from NG108-15 neuroblastoma-glioma cells stably transfected with HA-tagged D2L (IP). Mock immunoprecipitations were performed with control IgG. Blots were probed with anti-zDHHC4 or anti-SERZβ antibodies.
(TIF)

S2 Fig D2R agonist stimulation has no effect on the palmitoylation status of the receptor and palmitoylation of the D2R may not play a significant role in receptor signaling. (A) HEK-D2L cells were treated with 1 nm quinpirole for indicated times. Brackets indicate bands corresponding to D2R that were used for quantitation. Equal amounts of total cell lysate from each treatment were analyzed for phosphorylated ERK (pERK) and total ERK (tERK) by Western blotting (bottom two panels). Levels of palmitoylated D2L were normalized to the amount of immunoprecipitated receptor. The bar graph represents the average pixel density (as determined by ImageJ) from three separate experiments. Data was analyzed using a two-sided unpaired Student’s *t* test (expressed as ± SEM, *n* = 3). (B) FLAG-tagged WT-D2R or ΔC443-D2R cDNAs were transiently expressed in HEK-293T cells. Cells were treated with 1 nM of quinpirole for 5 minutes. Cell lysates were separated by SDS-PAGE and analyzed by Western blotting. Levels of pERK were normalized to levels of tERK. The bar graph represents the average pixel density (as determined by ImageJ) from four separate experiments. Data were analyzed using a two-sided unpaired Student’s *t* test (expressed as ± SEM, *n* = 4). (C) HEK-D2L cells were first treated with 100 μM 2-BP or ethanol, then with 50 μM forskolin or 50 μM forskolin + 10 μM dopamine for 15 minutes. cAMP levels were assayed using a colorimetric cAMP ELISA kit with acetylation. Levels of cAMP were normalized to levels of cAMP in forskolin treated cells. Data were analyzed using a two-sided unpaired Student’s *t* test (expressed as ± SEM, *n* = 3, *P < 0.05).
(TIF)
