# Supplementary figures and images for: Effect of C-Terminal S-Palmitoylation on D2 Dopamine Receptor Trafficking and Stability
Source: PLoS One. 2015 Nov 4;10(11):e0140661. doi: 10.1371/journal.pone.0140661 (PMC4633242; doi:10.1371/journal.pone.0140661)

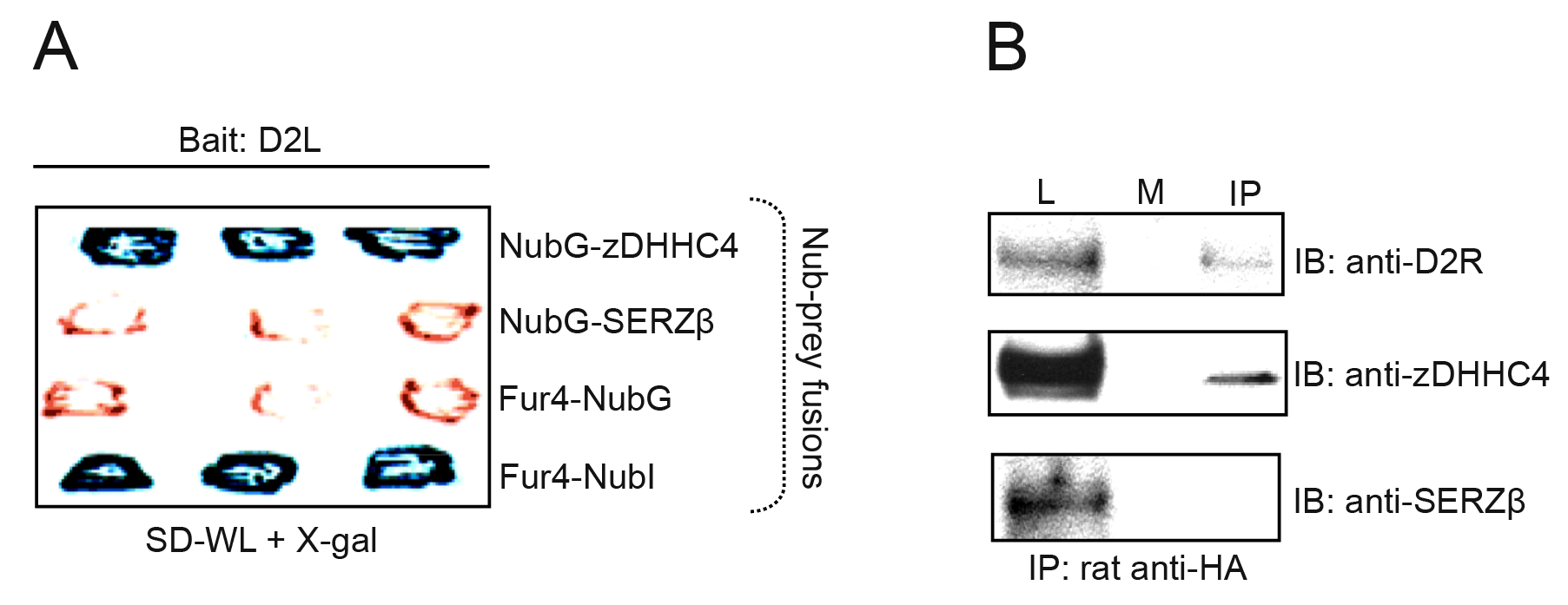

Supplement: S1 Fig — (A) MYTH assay in which yeast cells expressing human D2L were transformed with the following prey constructs: Fur4-NubG (negative control), Fur4-NubI (positive control), NubG-zDHHC4, and NubG-SERZβ. A positive interaction between D2L and NubG-zDHHC4 was detected, however, no interaction was observed with D2L and NubG-SERZβ, indicating that zDHHC4 is a D2L-specific interacting protein (blue yeast colonies). (B) HA-tagged D2L was immunoprecipitated from NG108-15 neuroblastoma-glioma cells stably transfected with HA-tagged D2L (IP). Mock immunoprecipitations were performed with control IgG. Blots were probed with anti-zDHHC4 or anti-SERZβ antibodies. (TIF) [file pone.0140661.s001.tif]

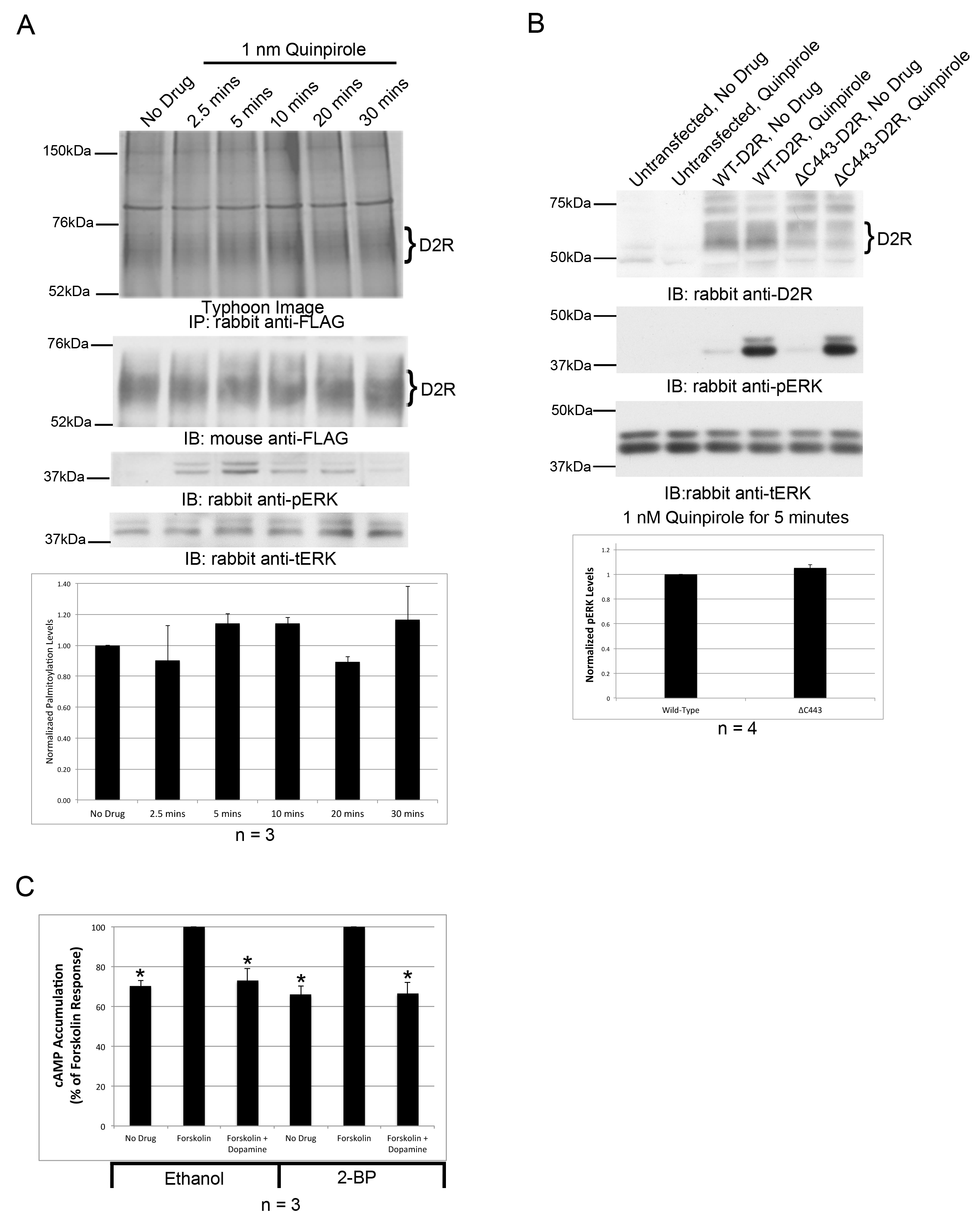

Supplement: S2 Fig — (A) HEK-D2L cells were treated with 1 nm quinpirole for indicated times. Brackets indicate bands corresponding to D2R that were used for quantitation. Equal amounts of total cell lysate from each treatment were analyzed for phosphorylated ERK (pERK) and total ERK (tERK) by Western blotting (bottom two panels). Levels of palmitoylated D2L were normalized to the amount of immunoprecipitated receptor. The bar graph represents the average pixel density (as determined by ImageJ) from three separate experiments. Data was analyzed using a two-sided unpaired Student’s t test (expressed as ± SEM, n = 3). (B) FLAG-tagged WT-D2R or ΔC443-D2R cDNAs were transiently expressed in HEK-293T cells. Cells were treated with 1 nM of quinpirole for 5 minutes. Cell lysates were separated by SDS-PAGE and analyzed by Western blotting. Levels of pERK were normalized to levels of tERK. The bar graph represents the average pixel density (as determined by ImageJ) from four separate experiments. Data were analyzed using a two-sided unpaired Student’s t test (expressed as ± SEM, n = 4). (C) HEK-D2L cells were first treated with 100 μM 2-BP or ethanol, then with 50 μM forskolin or 50 μM forskolin + 10 μM dopamine for 15 minutes. cAMP levels were assayed using a colorimetric cAMP ELISA kit with acetylation. Levels of cAMP were normalized to levels of cAMP in forskolin treated cells. Data were analyzed using a two-sided unpaired Student’s t test (expressed as ± SEM, n = 3, *P < 0.05). (TIF) [file pone.0140661.s002.tif]
